# Demod-CNN: A Robust Deep Learning Approach for Intelligent Reflecting Surface-Assisted Multiuser MIMO Communication

**DOI:** 10.3390/s22165971

**Published:** 2022-08-10

**Authors:** Mohammad Abrar Shakil Sejan, Md Habibur Rahman, Hyoung-Kyu Song

**Affiliations:** 1 Department of Information and Communication Engineering, Sejong University, Seoul 05006, Korea; 2 Department of Convergence Engineering for Intelligent Drone, Sejong University, Seoul 05006, Korea

**Keywords:** intelligent reflecting surface, demodulation, convolutional neural network, MIMO, OFDM

## Abstract

The intelligent reflecting surface (IRS) is a novel and innovative communication technology that aims at the control of the wireless environment. The IRS is considered as a promising technology for sixth-generation wireless communication. In the last few years, machine learning has emerged as a powerful tool for solving complex problems in diverse application areas. In this paper, we propose a convolutional neural network (CNN)-based demodulation technique called Demod-CNN in IRS-based wireless communication for multiple users. A multiple-input multiple-output based orthogonal multiple frequency division multiplexing system is considered for channel modeling. The received signal data are used for training and testing the model. The simulation results show that the proposed model performs better than the conventional demodulation technique.

## 1. Introduction

The intelligent reflecting surface (IRS) is considered as a novel smart radio technology for wireless communication systems [1]. The IRS is a two-dimensional metasurface of electromagnetic material that is composed of a large array of passive scattering elements with a specially designed physical structure [2]. Each element in the IRS can be controlled in a software-defined manner to change the electromagnetic proprieties (e.g., phase shift, amplitude) of the reflecting RF signal. This provides a unique advantage for non-line-of-sight communication to achieve a strong signal reception using the IRS [3]. One of the important properties of the IRS is that it can be implemented with low hardware cost and energy consumption for high gain beamforming [4,5,6]. Thus, it is desired to use the IRS in implementing millimeter wave communication for the sixth generation (6G). In addition, the IRS can be a potential candidate to enhance wireless sensor network (WSN) communication as it has the following advantages [7]: the IRS is composed of a nearly passive device electromagnetic material that is low cost, the IRS can reconfigure the wireless propagation environment to work as an alternative link when a direct link is not available, the IRS is energy-efficient compared to traditional relaying schemes, and the IRS can be implemented as full-duplex communication. Each of these features is desirable for WSN systems.

Multiple-input-multiple-output (MIMO)-based communication has been widely adopted in wireless communication [8,9]. MIMO-based communication has also been introduced to IRS-based communication [10,11]. Machine learning (ML)-based methods have also gained significant attention from researchers [12,13]. ML techniques have become very popular in solving complex problems for enhancing wireless communication systems without explicit programming [14]. A part of ML, deep learning (DL)-based schemes are also effective in predicting the channel estimation, beamforming optimization, and conducting phase optimization for IRS-based communication. The convolutional neural network (CNN) is a well known DL architecture that can provide effective solutions for different problem domains. In [15], authors proposed a denoising-convolution-neural-network-based channel estimation model for MIMO IRS. The transmitted complex data are received as a noisy image to increase the channel accuracy. The study in [16] proposed a twin CNN for the estimation of the direct channel and the cascade channel for IRS. The CNN network is trained with pilot data and can tolerate a user location of about 4 degrees. Another study in [17] used a CNN-based approach to estimate milimeter wave channel estimation. In addition to channel estimation, CNN can be applied in performance analysis such as bit error rate (BER) or symbol error rate (SER) for IRS-based communication.

Motivated from above, in this paper we propose a CNN-based ML architecture to improve the BER and SER performance of the MIMO-based IRS system. Orthogonal frequency division multiplexing (OFDM) based pilot symbols are transmitted through a frequency selective fading channel. Both IRS-based cascade channel model and direct channel are considered for communication. A CNN-based demodulation technique was proposed named Demod-CNN (Demodulation using CNN) to improve the channel performance in the case of MIMO communication. The proposed CNN model is trained with received complex data to map into bits for the channel in different signal-to-noise ratio (SNR) values. The trained model was tested with different datasets for performance evaluation.

The contributions of this study can be listed as:

- An IRS-based MIMO channel configuration system is considered for wireless communication to test machine learning assisted demodulation.
- A CNN-based demodulation technique Demod-CNN is proposed to demodulate the received signal.

*Notations*: The lower-case and upper-case boldface letter h and H represent a vector and matrix, respectively; $H^{H}$ denotes the conjugate transpose matrix of H; diag(x) denotes the diagonal matrix having vector x on its diagonal; and ⊗ is the Kronecker product.

## 2. System Model

### 2.1. OFDM Communication

OFDM is a multi carrier modulation technique applied in wireless communication for high spectral efficiency and good performance against frequency selective fading. The modulation process for OFDM starts by converting the binary inputs into phase shift Keying (PSK) or quadrature amplitude modulation for the purpose of mapping *D* parallel streams. *O* is the number of subcarriers in the OFDM system. If the value of *O* is increased, the performance of the model is degraded. Thus, the value of *O* should be chosen optimally depending on the required system. Let $X_{i}\left[q\right]$ be the *i*-th transmit symbol for the *q*-th subcarrier, where i=0,1,2,…,∞ and q=0,1,2,…,O−1. Next, the symbols are converted to the frequency domain from the time domain by using inverse fast fourier transform (IFFT). To avoid inter symbol interference, a cyclic prefix is added in the signal. One single symbol duration is considered as $T_{s}$, so for *Q*, symbol transmission requires $T_{sym}=QT_{s}$. Thus, for the *i*-th symbol and the *q*-th subcarrier, the OFDM signal $\Upsilon_{i,q}\left(t\right)$ is written as follows:

(1) $$\Upsilon_{i,q}\left(t\right)=\left\{\begin{array}{cc} e^{j2\pi f_{q}\left(t-iT_{sym}\right)}, & 0<t\leq T_{sym}\\ 0, & elsewhere, \end{array}\right.$$

where $f_{q}$ is the center frequency for the *q*-th subcarrier. The continuous time domain signal can be expressed as follows [18]:

(2) $$X_{i}\left(t\right)=\sum_{i=0}^{\infty }\sum_{q=0}^{O-1}X_{i}\left[q\right]e^{j2\pi f_{q}\left(t-iTsym\right)}.$$

The transmitted symbol can be considered as follows:

(3) $$X_{i}\left(n\right)=\sum_{q=0}^{O-1}X_{i}\left[q\right]e^{j2\pi qn/N}\mathrm{for}\;n\;=\;0,\;1,\;\ldots ,O-1,$$

where $f_{q}=q/T_{sym}$.

### 2.2. IRS Based Communication

**Figure 1:**
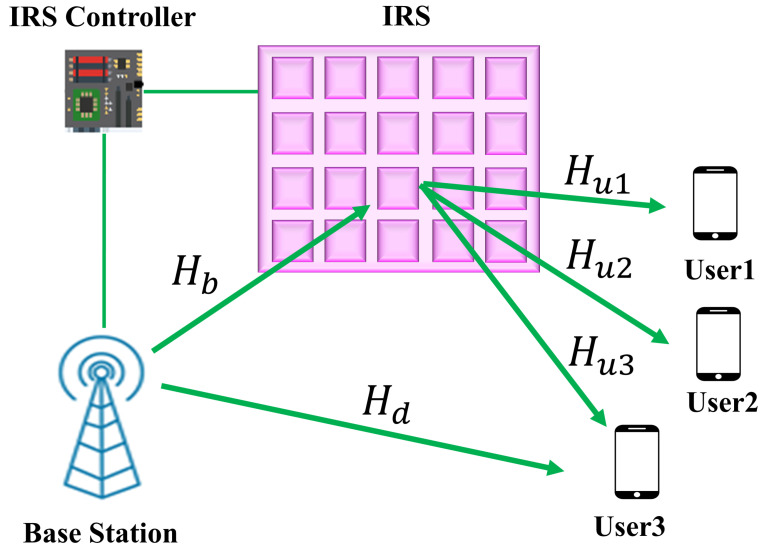
Intelligent reflecting surface in the multiuser MIMO communication system.

In this section, we describe the IRS network architecture and other related consideration. Figure 1 shows a communication scenario with the IRS where the base station (BS) has *M* uniform planner array (UPA) antenna and each user (UE) has a single antenna. The number of IRS elements is considered as *N*. The received signal via IRS for each UE can be expressed as follows [19]:

(4) $$y_{i}=H_{ui}^{H}\Psi H_{b}x+n,$$

where $y_{i}$ is the received signal at the *i*-th UE, the transmitted signal is $x\in C^{M\times 1}$, $H_{b}\in C^{N\times M}$ is the channel matrix between BS and IRS, $H_{ui}\in C^{1\times N}$ is the channel matrix from IRS to user *i*, and $n∼\mathrm{CN}(0,\sigma^{2})$ is the additive white Gaussian noise at the *i*-th user. Ψ is the diagonal matrix where $\Psi =diag\left(\xi \right)\in C^{N\times N}$ represents the phase shift values of IRS elements. Each element can be defined as $\xi =\left[\eta_{1}e^{j\omega_{1}},\eta_{2}e^{j\omega_{2}},\ldots ,\eta_{N}e^{j\omega_{N}}\right]\in C^{N\times 1}$, where $\eta_{n}\in \left[0,1\right]$ denotes the amplitude and $\omega_{n}\in \left[0,2\pi \right]$ is the phase shift coefficient of the *n*-th reflective element. Thus, Ψ can be written as:

(5) $$\Psi =\left[\begin{array}{cccc} \eta_{1}e^{j\omega_{1}} & \cdots & \cdots & \cdots \\ \cdots & \eta_{2}e^{j\omega_{2}} & \cdots & \cdots \\ \vdots & \vdots & \cdots & \vdots \\ \cdots & \cdots & \cdots & \eta_{N}e^{j\omega_{N}} \end{array}\right].$$

For easy calculation, the constant amplitude coefficient is considered as $\omega_{n}=1$. The total channel with direct communication link can be represented by [20]:

(6) $$y_{it}=H_{ui}^{H}\Psi H_{b}x+H_{d}x+n,$$

where $H_{d}\in C^{M\times 1}$ is the direct channel between BS and UE. The channel matrices $H_{ui}$, $H_{b}$, and $H_{d}$ follow the Rayleigh fading distribution, and each column *k* is modeled as:

(7a) $$\begin{array}{c} h_{ui(1,k)}=\kappa {\hat{h}}_{ui(1,k)} \end{array}$$

(7b) $$\begin{array}{c} h_{b(1,k)}=\kappa {\hat{h}}_{b(1,k)} \end{array}$$

(7c) $$\begin{array}{c} h_{d(1,k)}=\kappa {\hat{h}}_{d(1,k)}, \end{array}$$

where κ is the path loss factor and ${\hat{h}}_{ui(1,k)}$, ${\hat{h}}_{b(1,k)}$, and ${\hat{h}}_{d(1,k)}$ are $∼\mathrm{CN}(0,\sigma^{2})$. In addition, we consider the Saleh–Valenzuela channel model [8], which is applicable for a multipath propagation environment. The general theory of the Saleh–Valenzuela model for mmWave communication can be modeled as follows:

(8) $$h=\sqrt{\frac{N}{L}}\sum_{l=0}^{L}\alpha_{l}a\left(\gamma_{l}^{H},\phi_{l}^{H}\right),$$

where h is the channel vector, $\alpha_{l}$ is the complex gain of the *l*-th path, *L* is the total number of paths, $\gamma_{l}^{H}$ is the azimuth angle of departure, and $\phi_{l}^{H}$ is the elevation angle of departure and $a(\gamma_{l}^{H},\phi_{l}^{H})$ is the array response vector. For a typical $N_{1}\times N_{2}$ UPA, the array response vector written as follows [21]:

(9) $$a\left(\gamma ,\phi \right)=\frac{1}{\sqrt{N}}\left[e^{-j2\pi d\sin\left(\gamma \right)\cos\left(\phi \right)n_{1}/\lambda }\right]⊗\left[e^{-j2\pi d\sin\left(\phi \right)n_{2}/\lambda }\right],$$

where $n_{1}=\left[0,1,\ldots ,N_{1}-1\right]$ and $n_{2}=\left[0,1,\ldots ,N_{2}-1\right]$, λ is the carrier wavelength, and *d* is the antenna spacing fulfilling the condition d=λ/2. The BS-IRS channel $H_{b}$ can be expressed as follows:

(10) $$H_{b}=\sqrt{\frac{MN}{L_{1}}}\sum_{l_{1}=1}^{L_{1}}\beta_{l_{1}}b\left(\gamma_{l_{1}}^{H_{r}},\phi_{l_{1}}^{H_{r}}\right)a^{H}\left(\gamma_{l_{1}}^{H_{t}},\phi_{l_{1}}^{H_{t}}\right),$$

where $L_{1}$ represents the number of paths between the BS and the *i*-th UE, $\beta_{l}$ represents the complex gain of the paths, $b(\gamma_{l}^{H_{r}}\phi_{l}^{H_{r}})$ represents the steering vector related to the IRS, $a(\gamma_{l}^{H_{t}},\phi_{l}^{H_{t}})$ is the steering vector related to BS for the *l*-th path.

Next, the channel between the IRS and UE can be defined as follows:

(11) $$H_{ui}^{H}=\sqrt{\frac{N}{L_{2}}}\sum_{l_{2}=1}^{L_{2}}\beta_{l_{2}}a^{H}\left(\gamma_{l_{2}}^{H_{t}},\phi_{l_{2}}^{H_{t}}\right),$$

where $L_{2}$ is the number of paths between the IRS and the *i*-th user, $\beta_{l2}$ is the complex gain of paths, $\gamma_{l2}^{H_{t}}$ and $\phi_{l2}^{H_{t}}$ are the azimuth and elevation angle of departure of the signal, and $a(\gamma_{l_{2}}^{H_{t}},\phi_{l_{2}}^{H_{t}})$ is the steering vector. The cascade channel for BS to UE is as follows:

(12) $$\begin{array}{c} H_{ca}=\sqrt{\frac{MN}{L_{1}L_{1}}}\sum_{l_{1}=1}^{L_{1}}\sum_{l_{2}=1}^{L_{2}}\beta_{l_{1}}\beta_{l_{2}}diag\left(a^{H}\left(\gamma_{l_{2}}^{H_{t}},\phi_{l_{2}}^{H_{t}}\right)\right)\\ b\left(\gamma_{l_{1}}^{H_{r}},\phi_{l_{1}}^{H_{r}}\right)a^{H}\left(\gamma_{l_{1}}^{H_{t}},\phi_{l_{1}}^{H_{t}}\right). \end{array}$$

The channel matrix $H_{ui}^{H}\Psi H_{b}$ can be expressed as follows:

(13) $$H_{ui}^{H}\Psi H_{b}=H_{ui}^{H}diag\left(\xi \right)H_{b}=\xi^{T}diag\left(H_{ui}^{H}\right)H_{b}.$$

Then, the following equation is obtained:

(14) $$H_{ca}\xi^{T}=H_{ui}^{H}\Psi H_{b}.$$

The total received signal $y_{it}$ can be written as follows:

(15) $$y_{it}=\left(H_{ca}\xi^{T}+H_{d}\right)x+n.$$

### 2.3. Deep Learning Model

**Figure 2:**
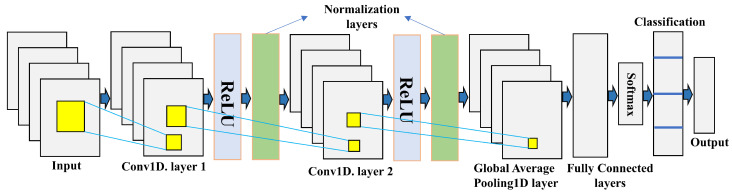
Deep learning architecture using CNN for IRS aided communication system.

Figure 2 shows the architectural design of the proposed CNN network. For successive training with the generated dataset, in this study, we have considered 1-D CNN model architecture to evaluate BER and SER for multiuser MIMO signals. The complex signal was first separated into real and imaginary parts. Then, the two numerical values along with the label are fed to the CNN model. In the proposed model, the input layer is fed into the OFDM data symbol, where the input size is equal to the number of features of input data. The size of the input features is considered as 2 × 2 × 2 = 8. The input size depends upon the number of users and the number of total antennas used in the system. The next two layers are connected by a convolutional 1D layer, ReLU activation function, and normalization layers. The first convolutional layer consists of a 3 × 3 filter size and total 32 numbers of filters are used. In the second convolutional layer, a 3 × 3 filter size, and 64 filters are used. The convolutional layer comprises a rectangular grid of neurons, where every neuron receives inputs from a rectangular part of the earlier layer. To reduce the output of the convolutional layers to a single vector, a global average pooling 1D layer is used. In the fully connected layer, each neuron is connected to all neurons in the previous layer and gathers all the features and internal information combined by the prior layers. In every time step of the CNN model, the fully connected layer works individually. We utilize the softmax activation function to derive the outputs for the final layer. In the terminal layer, we use the classification layer to map the output to vector probabilities and specify a fully connected layer with an output size matching the number of classes. The convolution operation for 1D forward propagation can be expressed as follows: [22]:

(16) $$\zeta_{k}^{v}=b_{k}^{v}+\sum_{p=1}^{N_{v-1}}\mathrm{conv}1D(w_{pk}^{v-1},s_{p}^{v-1}),$$

where the input is $\zeta_{k}^{v}$, the bias of the *k*-th neuron at layer *v* represents $b_{k}^{v}$, the output of the *p*-th neuron at layer (v−1) is defined as $s_{p}^{v-1}$, and the kernel from the *p*-th neuron at layer (v−1) to the *k*-th neuron at layer *v* presents $w_{pk}^{v-1}$. Hence, the dimension of the output arrays $s_{p}^{v-1}$ is greater than the dimension of the input array $\zeta_{k}^{v}$.

To normalize the *b*-th $\zeta_{k}^{v}$, we use the batch normalization layer [23] with the batch size *B*, which can be expressed as follows:

(17) $$\mu_{k}=\frac{1}{PVB}\sum_{p=1}^{P}\sum_{v=1}^{V}\sum_{b=1}^{B}{\left({\zeta_{k}^{v}}_{p}\right)}_{b},$$

(18) $$\sigma_{k}^{2}=\frac{1}{PVB}\sum_{p=1}^{P}\sum_{v=1}^{V}\sum_{b=1}^{B}({\left({\zeta_{k}^{v}}_{p}\right)}_{b}-\mu_{k}{)}^{2},$$

(19) $${\hat{\zeta }}_{k}^{v}=\frac{\zeta_{k}^{v}-\mu }{\sqrt{\sigma^{2}+\varepsilon }},$$

(20) $$\zeta_{k}^{v}=\alpha {\hat{\zeta }}_{k}^{v}+\varrho ,$$

where μ and $\sigma^{2}$ present the mean and variance of $\zeta_{k}^{v}$ respectively. *P* and *V* denote the size of the tensors for calculating the mean and variance. ε is a small constant, which is negligible. In the training process, α and ϱ are the learnable parameters, which will be updated. It can be noted that the learnable parameters α and ϱ denote the whole weights in convolutional layers, batch normalization layer, and fully-connected layer, respectively.

The activation function considered as the Relu function can be written as follows:

(21) $$f\left(x\right)=\left\{\begin{array}{cc} 0 & for\;x<0,\\ x & for\;x\geq 0. \end{array}\right.$$

To activate the output signal $\zeta_{k}^{v}$, herein, we utilize the Relu activation function, which can be formulated as follows:

(22) $${\zeta_{k}^{v}}_{R}=f\left({\zeta_{k}^{v}}_{R}\right).$$

In addition, to determine the possibility $\zeta_{k}$ of each category, we use the Softmax layer which can be expressed as follows:

(23) $$Softmax\left(\zeta_{k}\right)=\frac{\exp(\zeta_{fc\;k})}{\sum_{z=1}^{Z}\exp(\zeta_{fc\;z})}.$$

**Figure 3:**
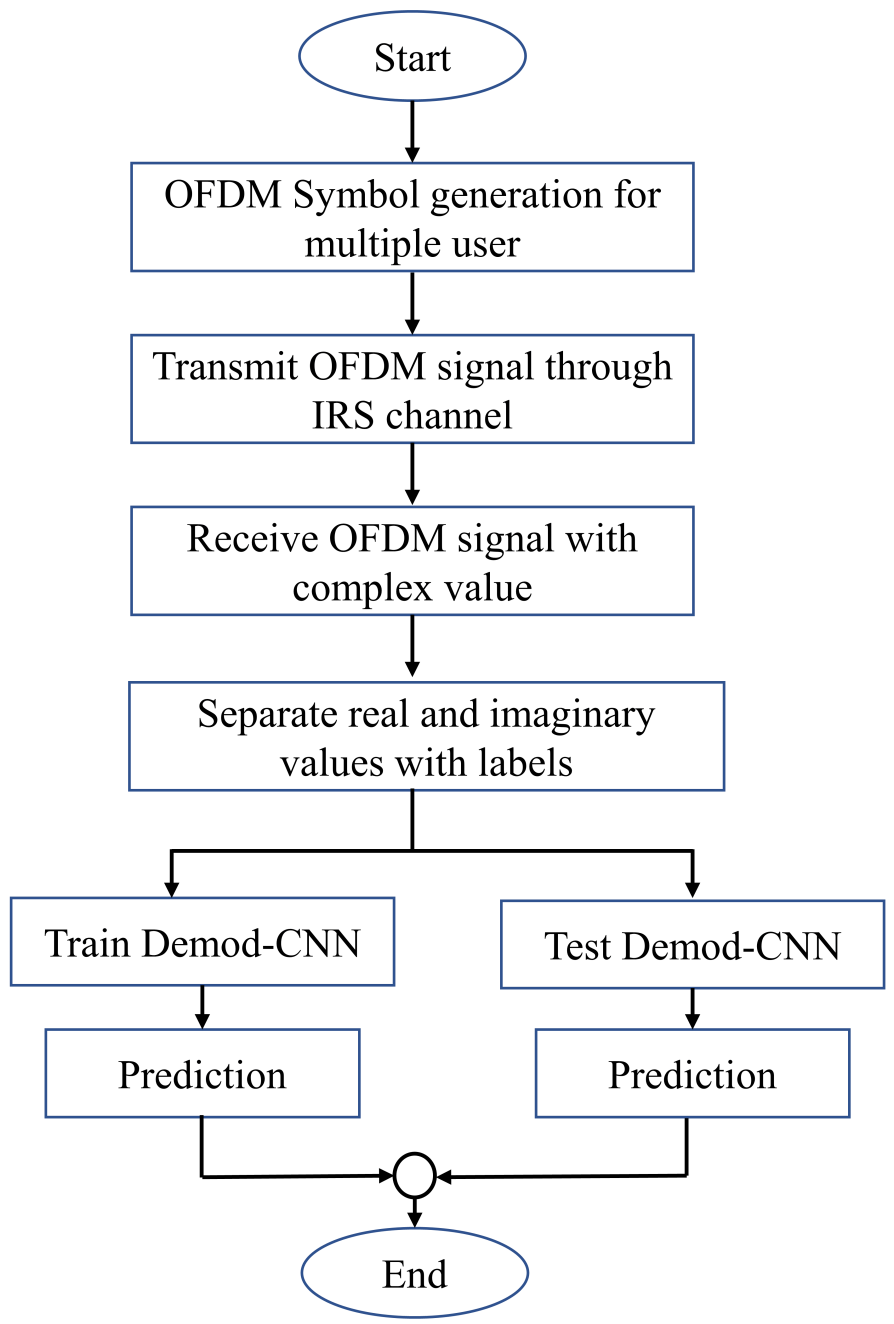
Flowchart for the proposed system workflow.

Finally, the mean-squared error (MSE) defines the loss function for the overall network, which is presented as follows:

(24) $$MSE=\sum_{i=1}^{N_{\vartheta }}{\left(\zeta_{k}-y_{it}\right)}^{2},$$

where $N_{\vartheta }$ represents the number of class labels. Figure 3 shows the overall flowchart for the proposed system.

The computational complexity of the proposed model can be specified as $O(P_{s}\times F_{s}\left(i_{c}\times f_{c}\times n_{c}\right)\times 2)$, where $P_{s}$ is the number of received input packets, $F_{s}$ is the OFDM block size, $i_{c}$ is the input size of CNN, $f_{c}$ is the filter size of CNN, and $n_{c}$ is the neuron size of CNN. In contrast, the conventional OFDM system has the complexity $O(M_{s})$, where $M_{s}$ is the modulation order [24]. The conventional OFDM is computationally efficient because it only uses IFFT and FFT. The complexity of the proposed model is higher than the conventional OFDM, but the performance is improved.

## 3. Simulation Setup

For the simulation, it is assumed that the number of BS antennas is *M* = 2 and the number of IRS elements is *N* = 512. It is assumed that the number of horizontal and vertical IRS elements are 32 and 16. Two users receive the data from the BS to UE = 2. The number of paths between BS and IRS is two, and the IRS to the *i*-th UE is four. For direct BS to UE channel, two paths are considered. In this simulation, mutltiuser interference is not considered, and it is assumed that each UE has same channel properties. Then, the BS can demodulate different UE signals successfully.We first determine the BER using the conventional technique following the same channel configuration for IRS-based communication. The simulation parameters are listed in Table 1. Additive white Gaussian noise is added to the transmission to simulate the channel noise environment. For the downlink channel, the three-channel matrix is considered: BS-IRS, IRS-UE, and BS-UE. The total $10^{6}$ packets are sent each time. One hundred and twenty-eight quadrature phase-shift keying (QPSK) symbols are generated using OFDM to estimate the channel error rate. To reduce the inter-symbol interference, a length of 32 cyclic prefix is added. For BER calculation, the data received by both users are considered simultaneously.

**Figure 4:**
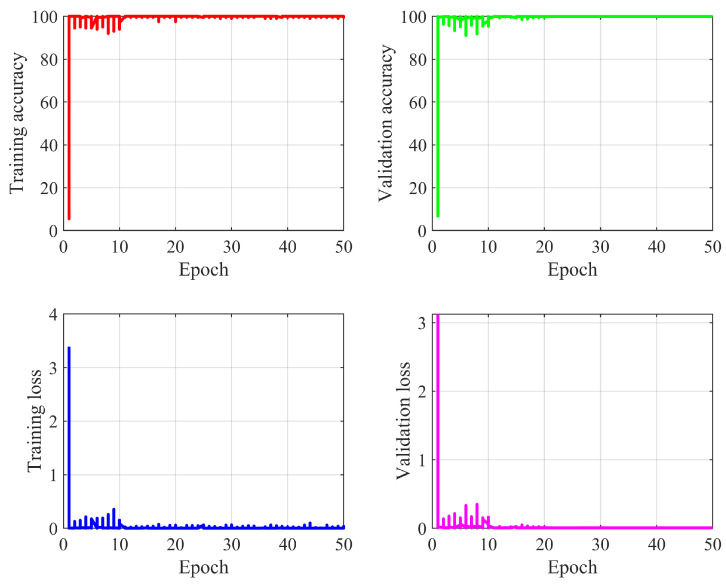
Proposed Demod-CNN model training and validation progress for 50 epochs.

The CNN-based model is trained by using the label and data generated by the QPSK symbol. The labeling is performed by combining the data from two transmitting antennas. As each antenna can generate 4 unique symbols, thus 4×4=16 labels are created for two data stream combinations. At the receiver, the received symbols are separated into real and imaginary parts along with the label. During the training, we capture data at the SNR 25 dB level to generate an offline input training set. We use 100,000 data sets for model development, of which (4/5) are used for training and (1/5) is used for validation. At 99.91% model accuracy, we stop the training process and save the model for inference. The training of the model less than 80,000 data sets does not produce the optimal performance. To achieve the highest accuracy, the model needs to have 80,000 data sets. However, 50,000 data sets can be employed for training, and in this case, the accuracy of the model is reduced. It is obvious that training with more data sets will require more time for training. Figure 4 shows the training and validation progress of the proposed model for 50 epochs. The upper left image shows the training accuracy, while the upper right image shows the validation accuracy. An aging lower left curve shows the training loss, and the lower right images shows the validation loss. It can be seen from the Figure 4 that after 20 epochs the model shows a stable performance, which indicates the model has learned the parameters from the data. If the number of antennas, users, IRS elements, OFDM parameters, or multi-paths is changed, the retraining of the model is required. In the future, we will try to build a model that can adopt these changes with minimum training.

## 4. Results and Discussion

**Figure 5:**
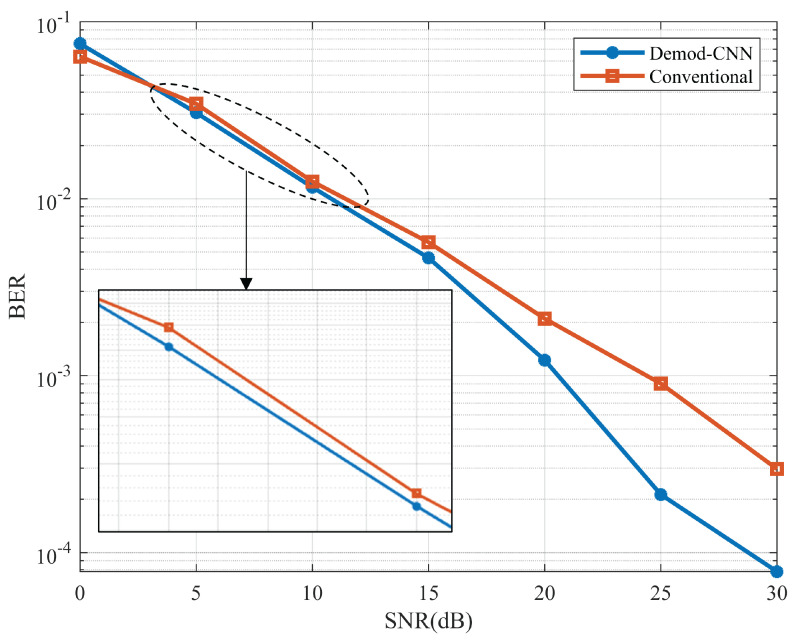
BER performance comparison of the proposed Demod-CNN and conventional technique.

**Figure 6:**
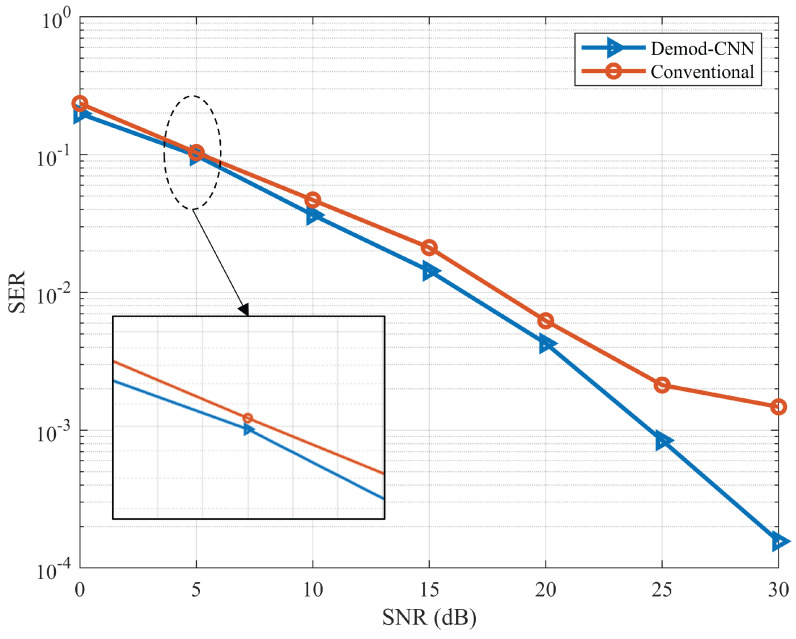
SER performance comparison of the proposed Demod-CNN and conventional technique.

In this section, the simulation results of the proposed model in terms of BER and SER are discussed. The BER and SER represent the average error rate for the two UEs. Both UEs data are first demodulated separately, and the error rate is calculated by error_rate=(user1+user2)/2. For BER, error_rate refers to the wrong demodulated bit at the receiver. For SER, error_rate refers to the wrong classification of the received symbol. In Figure 5, the BER curve is compared with the CNN-based demodulation against the conventional demodulation system. It is evident from the figure that CNN-based demodulation performs better than the traditional one. At SNR 0 dB, the model provides less accurate results than the conventional system because of the low SNR value. However, as the SNR values increase, the model performance improves. From SNR 5 dB, the model outperforms the conventional demodulation system. The BER for CNN-based demodulation has a similar trend to the conventional system up to 12 dB; after this point, BER improves dramatically for CNN-based demodulation. This indicates the efficient demodulation capability of the proposed CNN model. In Figure 6, SER versus SNR is plotted for the proposed model. The model shows better SER compared to the conventional demodulation scheme. After the SER 15 dB, the difference becomes clearer toward the higher SNR. Thus, the proposed CNN model can be used for improving BER and SER for IRS-based wireless communication.

## 5. Conclusions

In this paper, we have proposed a CNN-based model “Demod-CNN” OFDM demodulation system for IRS-aided multiuser MIMO communication. In the proposed model, we designed the input layer to receive the OFDM signal by separating real and imaginary parts of the symbol. It is then classified to recognize the transmitted bits by the classification layer. The simulation results provide that the proposed Demod-CNN can outperform the conventional demodulation system. In the future, we want to expand our model for multi-IRS systems to achieve massive MIMO communication.

## Figures and Tables

**Table 1:** Simulation parameters.

Parameters	Value
IRS elements	32 × 16
Transmitting antenna	2
Number of user	2
Number of subcarrier	128
Modulation	QPSK
Number of epoch	100
Minibatch size	200
Input size	8
Learning rate	0.01
Optimizer	ADAM
Noise	AWGN

## Data Availability

Not applicable.
